# Revisiting restricted reproductive rights in 2025: what do we need to know now?

**DOI:** 10.1017/ash.2025.56

**Published:** 2025-04-02

**Authors:** Pamela Bailey, Amy Crockett, Julie Ann Justo, Priya Nori

**Affiliations:** 1 Prisma Health Midlands, Division of Infectious Diseases, Columbia, SC, USA; 2 University of South Carolina, School of Medicine Columbia, Columbia, SC, USA; 3 Prisma Health Upstate, Department of Obstetrics and Gynecology, Greenville, SC, USA; 4 University of South Carolina, School of Medicine Greenville, Greenville, SC, USA; 5 Dartmouth Hitchcock Medical Center, Bennington, VT, USA; 6 Montefiore Health System, Albert Einstein College of Medicine, Bronx, NY, USA

## Abstract

In the aftermath of the 2022 *Dobbs v Jackson Women’s Health Organization* decision on access to reproductive healthcare, we published a commentary in this journal to inform the infectious diseases (ID) community about anticipated worsening of maternal and neonatal sepsis outcomes and relevant stewardship and healthcare associated infection issues. Three years later, we seek to keep the ID community engaged with important updates and intensify their commitment to providing high-quality care and reduce disparate health outcomes in this vulnerable population.

## Landscape of reproductive health in the US…Where are we now?

The United States continues to have the highest rate of maternal mortality of any high-income country; 80% deaths are considered preventable, and there are clear disparities in outcomes for non-white individuals.^1,2^ Infections, including sepsis and vaccine-preventable illnesses, are among the most common causes of maternal mortality.

Unacceptably high mortality rates in the U.S. existed prior to the 2022 *Dobbs v. Jackson Women’s Health Organization* decision by the Supreme Court to remove federal protections for abortion.^3^ Since that decision, 13 states have enacted total ban of all abortion procedures, an additional 28 states have passed bans with strict gestational age limits.^4^ Aside from concerns about these new barriers to care increasing the numbers of people seeking unsafe abortions, estimates project that a total abortion ban in the United States would increase pregnancy-related deaths from 675 to 724, with an additional 49 deaths annually. Non-Hispanic Black individuals would experience the greatest increase (33%), but all races and ethnicities would experience increases.^5,6^

Abortion bans were enacted within a tapestry of existing restrictions, including parental notification requirements, ultrasound requirements, and state-mandated waiting periods. Furthermore, many of these bans have vaguely worded exemptions for risks to maternal health, serious fetal anomalies, and pregnancies resulting from rape or incest. Confusion regarding the status of laws at the state or local level and ambiguity of the legislative language leaves providers uncertain about the type and timing of medical care they can lawfully provide. Conditions of concern include treatment of ectopic pregnancies, care for rupture of membranes before fetal viability, or care for incomplete spontaneous abortions (“miscarriage”). While stressful for healthcare providers, patients note “feeling hurt and confused” when trying to navigate medical care for reproductive health, especially after receiving news of lethal fetal conditions.^7^

Confusion among both providers and patients impedes timely medical care if a medical crisis arises during pregnancy. Timely treatment within the “golden hour,” is as critical for maternal sepsis as it is for other serious medical conditions.^8^ The legal quagmire around reproductive healthcare means time ticks by while patients receive no or suboptimal care and experience increased complications—as predicted.^1,9^

## What should the infectious diseases community expect?

In the post-Dobbs era, ID specialists may encounter more patients with infectious complications of pregnancy. Maternal and infant morbidity and mortality are significantly impacted by access to obstetric care, particularly for rural residents and racially minoritized women; (42.4%) of all hospitals and (52.4%) of rural hospitals did not have obstetric care in 2022.^10^ Downstream delays in treatment of obstetric complications such as maternal sepsis are expected to worsen if obstetric care is inaccessible. Timely treatment by emergency medicine, internal medicine, and ID specialists provides a critical linkage to care. For this reason, ID specialists must familiarize themselves with local laws and exemptions for patients in crisis seeking reproductive healthcare, as well as up-to-date recommendations, like ACOG’s liberalization of initiation of antibiotics if chorioamnionitis is suspected.^11^ Additionally, ID specialists working in institutions without an in-house obstetric service can develop institutional “sepsis in pregnancy” pathways to expedite care.

The ID specialty has long served as a primary advocate for vaccines, and we must continue to advocate for protecting pregnant patients and providing passive immunity for neonates. National trends of decreasing confidence in vaccines have also touched obstetric patients, particularly as COVID-19 and RSV vaccines were added to the 3^rd^ trimester recommendations.^12^ Compounding the harm are record high numbers of pertussis cases in 2024 compared to prior years.^13^ ID specialists must continue to lead conversations on vaccines as a public health priority.

Another preventable condition with an unfortunate resurgence is congenital syphilis. Between 2012 and 2021, congenital syphilis cases increased (755%) in the United States, to 3,761 cases in 2022 with 231 (6%) stillbirths and 51 (1%) infant deaths.^14^ Lack of timely testing and adequate treatment contributed to almost (90%) congenital syphilis cases in the United States—across all regions and racial/ethnic groups.^14^ ID specialists play a pivotal role in the timely diagnosis and treatment of maternal and congenital syphilis.

## Call to action

### Ensuring trust in public health, vaccines, and preventive therapies by harnessing provider-patient relationships

While confidence in public health entities has eroded, (95%) of both Democrats and Republicans reported trusting their personal physicians to provide recommendations about health issues.^15^ Healthcare providers’ vaccination attitudes are impacted by anticipated patient/parental hesitancy, lacking clear vaccination guidelines, time constraints, and concerns about cost. Provider hesitancy also stems from inadequate knowledge, low vaccine confidence, and suboptimal uptake themselves.^16^ These are critical areas to address to reinforce the “trusted messenger” status of personal physicians.

Consistent reiteration of favorable post-marketing safety data, especially for newer vaccines such as RSVpreF, is important for maternal and fetal health, particularly when nirsevimab may be in short supply.^17^ Additionally, emphasizing adverse consequences of vaccine-preventable infections for both mother and baby are critical for ensuring optimal health decisions.^18^ Trusted provider-patient relationships should also be harnessed to educate patients on new and old vaccines and therapies.

### Ensuring standard of care management of maternal sepsis

Maternal sepsis is critical to recognize and treat expeditiously; however, our understanding of the microbiology of these infections is antiquated.^19,20^ Source control is a must, yet pregnant persons may be medically managed if surgical management is delayed while navigating the legalities.^11,21^ Notably, a survey from 2012 noted major inconsistencies among OB/GYN providers in terms of diagnostic criteria, antimicrobial selection, and duration of antimicrobials for chorioamnionitis.^22^ In fact, depending on group B *Streptococcus* (GBS) status, mode of delivery (laboring Cesarean section or not), surgical site infection prophylaxis, and penicillin allergies, there are approximately 25 endorsed ACOG regimens for chorioamnionitis management, contributing to an ongoing lack of clarity. We desperately need guideline-endorsed, evidence-based alternatives to traditional “triple therapy” of ampicillin, gentamicin, ± clindamycin, such as cefoxitin or piperacillin/tazobactam.^23,24^ Further research on ideal antimicrobial selection and duration, and appropriate diagnostics to distinguish infection from non-infectious causes of fever is critical.

In some institutions, the 2023 intravenous clindamycin shortage led to a streamlining and updating of historic “triple therapy” in favor of cefoxitin for chorioamnionitis and endometritis.^23^ This upgrade is evidence-based, dictated by local microbiology, logistically more efficient, and likely less toxic with favorable outcomes.

### Advocating for pregnant patients from minoritized communities

As a whole, pregnant and lactating persons are more likely to be excluded from clinical research, though efforts are now underway to include these “scientifically complex” (rather than “vulnerable”) persons in research.^25^ Reduction of health disparities is a complex public health and public policy issue, involving pharmacoequity and equitable access.^26^ This will remain a struggle as reproductive care deserts expand and patients remain unaware of their options.

### Ensuring providers understand current abortion laws in their state

**Figure 1.**
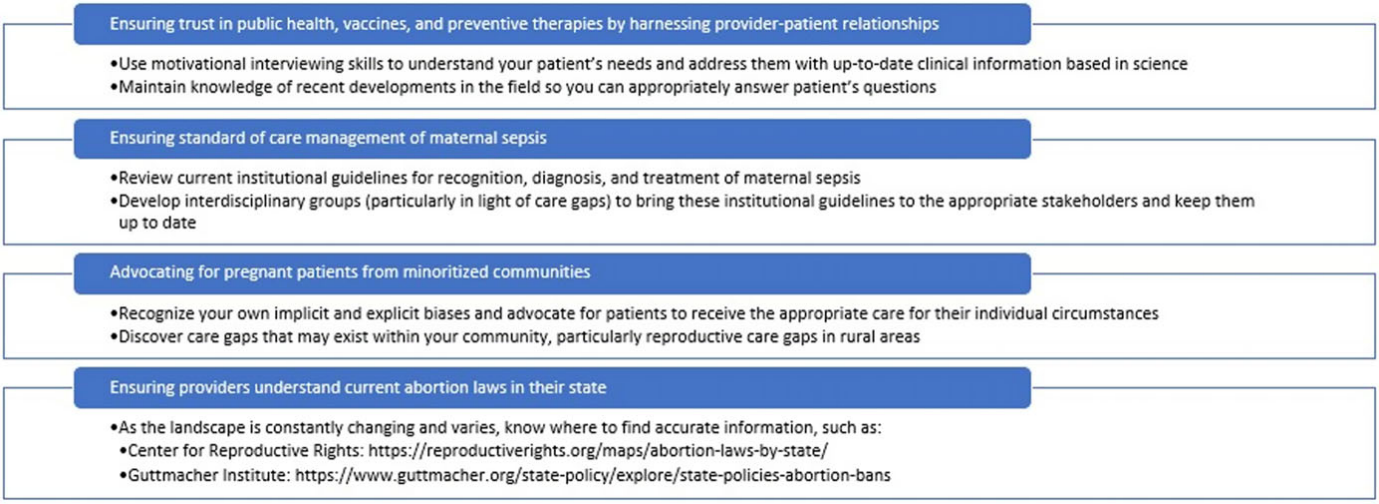
Action items for infectious diseases clinicians.

To best counsel our patients on their healthcare options, we must stay up to date on the changing legal restrictions at the federal, state, and local level (Figure 1). We must appreciate complex obstetric access issues in rural versus urban areas of the U.S. and be well-versed in newer telehealth modalities for accessing reproductive care in light of the medicolegal complexities.^27^

## Conclusion

Since the landmark 2022 Dobbs decision, restrictions on reproductive healthcare continue to evolve. We compel ID providers to remain up to date on (1) poor maternal–fetal health statistics in the U.S. and socioeconomic factors contributing worsening outcomes, (2) policies restricting and enabling access to care, (3) evidence-based antibiotic regimens for sepsis in pregnancy, (4) recommended vaccines and preventive therapies for mother and baby, and (5) effective strategies to communicate health information as trusted healthcare providers.
